# Redefining the meaning of dementia: A qualitative study of meaning, stigma, and care experiences among family and professional carers

**DOI:** 10.1002/dad2.70521

**Published:** 2026-10-21

**Authors:** Anna Tjin, I Made Pasek Saradahtam Bendesa, Robert Stewart, Thang Leng Leng

**Affiliations:** ^1^ Psychological Medicine Institute of Psychiatry Psychology & Neuroscience King's College London London UK; ^2^ Department of Japanese Studies National University of Singapore Singapore Singapore; ^3^ Department of War Studies Faculty of Social Science & Public Policy King's College London London UK; ^4^ South London and Maudsley NHS Foundation Trust London UK

**Keywords:** caregiving, coping mechanism, dementia, diagnosis, qualitative, stigma

## Abstract

**INTRODUCTION:**

As dementia prevalence rises, reliance on carers increases substantially, yet little is known about how carers experience, interpret, and redefine dementia. This qualitative study explored how family and professional carers in the United Kingdom (UK) make sense of dementia, focusing on the meanings they ascribe and how stigma shapes care practices, trajectories, and preferences within cultural, moral, and structural contexts.

**METHODS:**

Purposive sampling recruited 12 family carers and three professional carers across the United Kingdom, and data were analyzed using inductive reflexive thematic analysis. The perspectives of professional carers were included as contextual insights into dementia caregiving experiences rather than for direct comparison with family carers.

**RESULTS:**

Carers described dementia caregiving as a totalising, unpredictable process requiring ongoing reconfiguration of daily life, family roles, and care expectations. Disclosure, reasoning with symptoms, and care‐setting decisions were shaped by cultural, moral, and structural factors, balancing practical needs, autonomy, public responses, and emotional wellbeing.

**DISCUSSION:**

Recognizing the dynamic processes of dementia caregiving within the United Kingdom care context underscores the need for iterative support systems that promote the resilience and quality of life for carers and people with dementia.

## Highlights

- Explored experiences of family & professional carers using qualitative methods
- Identified “Re–” meta‐theme capturing iterative nature of caregiving and five themes
- Stigma shaped disclosure, care decisions, and caregiving strategies across contexts
- Carers balanced cultural, moral, & structural constraints while supporting autonomy
- Adaptive support systems are needed to improve carer preparedness and wellbeing

## 1 BACKGROUND

Dementia presents considerable challenges for families, communities, and health systems worldwide.^1^ Progressive cognitive and behavioral symptoms,^2^ together with increasing frailty and comorbidities,^3^ contribute to dementia being a leading cause of disability and dependency globally.^1^ The growing prevalence of dementia implies a parallel reliance on carers, yet the global scale of caregiving remains poorly quantified. Estimates vary widely, from approximately 40 million full‐time equivalent carers in 2015 to a projected 65 million by 2030,^4^ corresponding to an estimated 1,612^5^ to 2,089 hours per carer per year.^4^ In the United Kingdom (UK), approximately 1.8 million individuals provide unpaid care for people with dementia, contributing an estimated £21.1 billion annually through foregone employment and lost productivity.^6^, ^7^ This scale of caregiving underscores not only its economic value but also the important role of carers in sustaining long‐term care within the UK health and social care system, particularly amid a persistent structural divide between health and social care.^8^, ^9^

Evidence suggests that this global reliance affects individuals across diverse settings,^10^ with differing consequences for family and professional carers.^11^ While caregiving can foster personal growth and a sense of purpose,^12^ dementia often necessitates complex, sustained care, including difficult decisions such as institutionalization or sacrificing employment and social roles.^10^ Carers and care recipients also contend with social stigma,^13^, ^14^ conceptualized as “the co‐occurrence of labeling, stereotyping, separation, status loss, and discrimination^15^”, which can delay diagnosis, reduce support, and compromise quality of life.^16^ Despite increased public awareness, misconceptions persist; a 2024 global survey conducted by Alzheimer's Disease International indicated that around 30% of respondents perceived dementia as dangerous, and 40% attributed it to family neglect.^17^

Existing studies rarely capture both family and professional perspectives, leaving gaps in understanding where stigma persists, how it shapes care practices, and where supports may be most effective. Qualitative approaches are well‐suited to exploring these complex, context‐dependent experiences, illuminating how cultural, social, and structural factors within specific care contexts shape caregiving practices and perceptions of dementia.^18^ This study aims to explore how family and professional carers in the United Kingdom make sense of dementia, the meanings they ascribe to it, their experiences of stigma, and how these experiences influence care practices, trajectories, and preferences within cultural, moral, and structural contexts shaping dementia care.

## 2 METHODS

This study formed part of a larger research programme titled “Access, Barriers, and Facilitators of Dementia Care (ABCDem)”, examining barriers and facilitators to dementia care across the United Kingdom and Singapore. Participants were recruited through the South London and Maudsley Consent for Contact register and the King's College London research register. Individuals who had consented to future research contact were identified and referred to the research team. The researcher provided study information and assessed capacity to consent. Some eligible individuals did not respond to initial contact; no eligible participants actively declined participation. Eligible participants were adults (≥18 years) providing care for individuals formally diagnosed with dementia, able to give informed consent, and with current or prior first‐hand experience supporting access to dementia‐related healthcare services in the United Kingdom. For this study, *family carers* were defined as unpaid relatives serving as the primary carers, and *professional carers* as paid care staff employed by agencies or nursing homes providing dementia care in community or residential settings. Professional carers provided contextual perspectives on dementia care provision and service experiences; the study was not designed for comparison between caregiver groups.

Purposive sampling was informed by demographic diversity reported by the Office for National Statistics^19^ and our population‐level study,^20^ ensuring variation in gender, ethnicity, dementia subtype and stage, and caregiving type. Sampling prioritized family carers to reflect the study focus on lived caregiving experiences, while professional carers provided complementary perspectives. Recruitment and data collection occurred between June and December 2025 through semi‐structured individual interviews (60–90 minutes), conducted face‐to‐face, online, or by telephone. Interviews were conducted by the first author (A.T.), a female PhD researcher trained in qualitative methods. A.T. had no prior relationship with participants. Reflexive field notes were maintained, and interpretations were discussed within the research team throughout the analytic process. The interview guide was developed collaboratively with medical professionals, dementia organization representatives, people with dementia, and family carers, and was iteratively refined after each interview. This semi‐structured approach^21^ provided a framework for discussion while allowing participants to explore their own perspectives. Early interviews were broad and exploratory, with later interviews refined to probe emerging themes. Data sufficiency was assessed in terms of richness and complexity, and the research team regularly considered sample size and practical feasibility throughout data collection. The study is reported in accordance with the Consolidated Criteria for Reporting Qualitative Research.^22^

**RESEARCH IN CONTEXT**

1. **Systematic review**: Previous studies have often focused on consequences of caregiving. Few have examined how carers actively reason with dementia symptoms, negotiate disclosure, or reconfigure care across cultural, generational, and structural contexts. Carers’ perspectives across diverse ethnicities, genders, dementia stages, and subtypes remain underexplored.
2. **Interpretation**: Carers draw on cultural, spiritual, and moral frameworks to interpret dementia, manage symptoms, disclose dementia, and cope with demands and expectations. Caregiving involves continuous adaptation of daily routines, family roles, and care expectations, reflecting emotional, ethical, and practical complexities. Carers’ strategies are shaped by individual, familial, and societal influences, highlighting the dynamic, context‐dependent nature of sustaining care.
3. **Future directions**: Future research should evaluate interventions that integrate carers into dementia care pathways, assess long‐term effects of disclosure strategies, and compare experiences across healthcare systems. Understanding how cultural norms, structural constraints, and family dynamics influence caregiving can inform policies and services to support diverse carers effectively.

### 2.1 Data analysis

Guided by a constructivist qualitative paradigm, this study employed reflexive thematic analysis using an inductive approach,^23^ conducted concurrently with data collection to allow iterative comparison of emerging concepts with new data. This approach enabled understanding phenomena in context, linking concepts and behaviors while ensuring findings were data‐driven and acknowledging researcher positionality. Methodological reflexivity^24^ was applied throughout, with team members critically examining their own paradigmatic orientations and potential influence on the research. Analysis was conducted both individually and collaboratively to identify and mitigate bias. The research team, comprising a psychiatrist, mental health researchers, and an anthropologist, discussed preconceptions iteratively throughout data interpretation, treating findings as co‐constructed yet data‐driven.

All interviews were audio‐recorded, transcribed verbatim, and checked for accuracy against the recordings. Reflexive thematic analysis followed standard steps^23^: familiarization with the dataset, coding, generating initial themes, reviewing and refining themes, naming themes, and write‐up. Authors engaged with transcripts, audio recordings, and analytic memos to understand the data.

Initial coding was undertaken in NVivo by two researchers to support reflexive engagement with the data. Codes and interpretations were discussed within the research team to challenge assumptions, enhance reflexivity, and develop themes. Themes were iteratively refined through ongoing engagement with the dataset to produce a coherent account of participants’ experiences.

## 3 RESULTS

The final sample included 15 participants: 12 family carers and three professional carers (Table 1).

**TABLE 1:** Carers’ characteristics.

No.	Caregiving type	Relationship	Age	Gender	Ethnicity	Care recipient	Dementia type	Stage
1	Family	Spouse	73	Female	White British	Husband	VD	Mild
2	Family	Spouse	68	Female	Asian/Asian British	Husband	VD & AD	Mild
3	Family	Son	58	Male	Asian/Asian British	Mother	AD	Mild
4	Family	Daughter	42	Female	White European	Mother	AD	Moderate
5	Family	Daughter	49	Female	White British	Mother	VD	Moderate
6	Family	Son	57	Male	White British	Father	VD	Moderate
7	Family	Son	65	Male	Black/Black British	Father	VD	Moderate
8	Family	Grandson	28	Male	Asian/Asian British	Grandmother	AD	Severe
9	Professional	Professional	52	Female	Mixed race	Patients	NOS	Severe
10	Professional	Professional	31	Male	Black/Black British	Patients	NOS	Severe
11	Professional	Professional	35	Female	Black/Black British	Patients	NOS	Severe
12	Family	Daughter	55	Female	White British	Father	AD	Deceased
13	Family	Son	42	Male	White British	Father	AD	Deceased
14	Family	Son	59	Male	Asian/Asian British	Mother	AD	Deceased
15	Family	Spouse	61	Female	Black/Black British	Husband	AD & PDD	Deceased

**^Abbreviations: AD,^ :**Alzheimer's disease; NOS, not otherwise specified; PDD, Parkinson's disease dementia; VD, vascular dementia.

The overarching “Re–” meta‐theme captured the continuous adjustment, reinterpretation, and renegotiation through which carers made sense of dementia over time. It provided a framework for understanding caregiving as an evolving process rather than a single transition, with carers repeatedly revisiting meanings, expectations, and responses. Across the five themes, carers described reinterpreting dementia, negotiating stigma, and adapting care practices as circumstances changed.

Carers characteristics: Professional carer (Pro); Female (F), Male (M).

Dementia type and severity: Alzheimer's disease (AD), vascular dementia (VD), Parkinson's disease dementia (PDD), not otherwise specified (NOS), mild (Mid), moderate (Mod), severe (Sev), deceased (Dec).

### 3.1 Re‐evaluating memories and meaning of dementia

This theme captures how dementia became personally meaningful during the transition into caregiving. Before caregiving roles, dementia was encountered in passing, through media or social discourse, with little personal relevance or emotional weight. Carers describe it as “a word in the dictionary” (Son, Mid‐AD) or “something that happens to somebody else” (Daughter, Dec‐AD). Dementia existed as an intellectual abstraction rather than a lived reality: “I knew very little about dementia because it didn't affect me” (Pro‐F, NOS). In some cases, the condition was unrecognized entirely within social and familial environments, equating dementia to “dragons or unicorns” (Pro‐M, NOS).

Some carers acknowledged that dementia had long existed within their communities, but that it was often euphemized or socially minimized. This perception shifted with caregiving, as behaviors previously normalized or culturally tolerated were retrospectively recognized as indicators of cognitive decline. One carer reflected:

“I recall conversations with a friend when she described a crazy family member. Now, I think they might have dementia.” (Wife, Mid‐VD&AD)

Similarly, anecdotes of forgetfulness or confusion, for relatives who “lost their marbles” (Daughter, Mod‐AD) or “got muddled with their thoughts” (Pro‐M, NOS) were recognized only in hindsight as potential dementia symptoms. This re‐evaluation also prompted carers to reflect on the presence of dementia within their immediate surroundings. One participant reflected, “I feel like dementia is not something new, we just swept it under the rug” (Son, Mod‐VD), while another observed that the term's visibility had increased: “Dementia has now become a common word…even if someone doesn't understand it, they know it's bad.” (Wife, Dec‐AD&PDD)

Carers described a growing awareness of the gap between “knowing about dementia and living with it” (Son, Dec‐AD), acknowledging the limits of their assumptions about resilience:

“If they told me about dementia before, I wouldn't believe them…Nothing prepared me for this…” (Grandson, Sev‐AD). These accounts reveal retrospective meaning‐making, as carers reinterpreted earlier experiences and recognized how limited awareness shaped preparedness.

### 3.2 Realigning acceptance and realities around diagnosis

This theme focuses on how formal diagnosis reshaped carers’ expectations, emotions, and responses to dementia. Learning that the person they cared for had received a dementia diagnosis represented a pivotal yet internally contradictory moment for carers. Diagnosis confirmed suspicions that had gradually formed, crystallizing observations previously noted but not formally named. One carer reflected, “it's what everyone was already thinking” (Wife, Mid‐VD), while another described it as “it is finally verbalized” (Son, Mod‐VD). Rather than resolving uncertainty, diagnosis compelled carers to confront a reality that had been partially ambiguous and emotionally buffered.

Carers described divergent responses to this confirmation. Some adopted a pragmatic, action‐oriented stance, emphasizing preparedness: “I need to face the music, before things get worse” (Daughter, Mod‐VD), or “I had to face the reality, this is the reality” (Son, Dec‐AD). Others articulated an acceptance‐oriented approach: “there is no cure, but there are things that make it easier” (Son, Mod‐VD). Some framed dementia within broader population ageing, noting that “most people will end up with dementia” (Grandson, Sev‐AD). Conversely, the diagnosis intensified despair, particularly regarding incurability: “There is no light at the end of the tunnel” (Son, Mid‐AD) or “No cure, and there is nothing they can do for the family” (Wife, Dec‐AD&PDD).

Acceptance was prolonged, shaped by dementia meanings, identity, pride, and fears of devaluation. The care recipient's lifelong values of self‐reliance and perfection complicated adjustment: “She wouldn't accept that she could be anything other than normal” (Daughter, Mod‐AD). The diagnosis was experienced as harmful labeling “because of her perception of what dementia is” (Son, Dec‐AD). Dementia was frequently equated with mental illness and social exclusion, with care recipients reportedly “rather have cancer, because it's a real illness” (Daughter, Dec‐AD). Dementia was perceived as “crazy” (Son, Mid‐AD) and associated with “locked away” (Son, Mid‐AD) or “cast out” (Wife, Dec‐AD&PDD). These perceptions contributed to refusals of care, even at considerable cost to comfort and dignity, as accepting support was seen to “invite pity and embarrassment” (Son, Mid‐AD).

Professional carers described a parallel dilemma, balancing respect for autonomy with the ethical consequences of disclosure. Daily care required ongoing negotiation, as professionals were repeatedly asked “a million times a day” (Pro‐M, NOS) on why residents were in care. Disclosure became a situational judgment rather than a one‐time event, requiring carers to weigh honesty against emotional protection: “Not everyone can accept it. Some can, some won't” (Pro‐F, NOS). Diagnosis was not a definitive moment of acceptance but an ongoing negotiation between medical recognition, identity, and stigma.

### 3.3 Reasoning with symptoms

This theme focuses on how carers interpreted and explained dementia‐related changes, rather than their acceptance of diagnosis or adjustment to the caregiving role. Carers described ongoing interpretative work to understand dementia, particularly when medical explanations were emotionally insufficient or difficult to integrate into existing belief systems. Spiritual explanations represented individual meaning‐making strategies used by some carers alongside or in tension with biomedical understandings. Some drew on religious or spiritual narratives to explain why dementia occurred and how it should be understood, described as “putting spiritual beliefs wherever they can see fit” (Grandson, Sev‐AD). Dementia was sometimes understood through karmic narratives, described as “spirits from a previous life came to ruin this life” (Wife, Mid‐VD&AD), occasionally resulting in moralized interpretations of causation: “it's because she did things in her past life” (Grandson, Sev‐AD). Others described religious frameworks that conflicted with biomedical understandings, particularly where illness challenged beliefs about divine benevolence: “Only good things come from God… since dementia is bad it's from the devil” (Son, Mod‐VD). This contrast was captured by one professional carer: “The church will tell you good things, while the hospital tells you bad things” (Pro‐F, NOS).

Importantly, spiritual reasoning did not exclude medical care. Many carers described a dual explanatory approach, in which medical treatment addressed symptoms while faith‐based understandings helped carers tolerate uncertainty: “you should go through the health care but then put your faith on God to make it work” (Son, Mod‐VD). In some contexts, spiritual explanations were also described as more accessible, particularly where financial constraints limited access to services: “The church are free, medical and social care are not” (Son, Dec‐AD).

Reasoning also shaped interpretation of emerging symptoms. Memory loss and confusion were often normalized as ageing or physical decline: “something old people do” (Wife, Dec‐AD&PDD), or “it's because of his eyesight” (Daughter, Dec‐AD). These interpretations functioned as emotional buffers, delaying confrontation with dementia until disrupted by critical incidents: “she got lost on the way home… that's when I knew this is serious” (Daughter, Mod‐AD), or “we almost had a fire because he forgot to turn off the kettle” (Wife, Mid‐VD). Carers described the difficulty of interpreting symptoms, particularly when behaviors fluctuated, making it challenging to distinguish intentional actions from illness‐related changes: “she was fine yesterday and not today, I don't know if it's her or dementia” (Son, Mid‐AD). While attributing behaviors to dementia helped some carers manage distress, emotional strain often persisted: “I know it's dementia, but I still get angry… I just can't help it” (Wife, Mid‐VD). Carers constructed explanations for dementia through multiple knowledge systems to manage uncertainty and maintain coherence when symptoms challenged their understanding of the person.

### 3.4 Revealing and relabeling dementia to the public

This theme examines how carers managed dementia's meaning in interactions with others through disclosure and concealment. Carers described disclosure of dementia as an ongoing, situationally negotiated process rather than a single decision. Choices about whether to reveal or conceal the diagnosis were shaped by anticipated social reactions, moral considerations, and the perceived legitimacy of dementia relative to other conditions. Dementia was frequently described as more socially recognizable than mental health conditions, such as depression. Some carers noted that “dementia is more normal than depression” (Daughter, Dec‐AD), contrasting it with depression being dismissed as “not real” (Pro‐F, NOS) or “just stress” (Son, Mod‐VD). Dementia, by contrast, was framed as objectively verifiable and biomedical: “dementia is real, you can see them in scans” (Daughter, Dec‐AD). This perceived legitimacy shaped carers’ expectations of public understanding and reduced concerns about disbelief.

The decision to disclose was often driven by the visibility of dementia‐related changes. Carers emphasized that symptoms were difficult to conceal in everyday interactions: “dementia isn't something you can hide… people notice straight away” (Wife, Mid‐VD) and “you would know something is wrong within 5 minutes” (Son, Mod‐VD). Concealment was therefore seen as a strategy to manage social interactions and reframe public interpretations of behavior rather than a voluntary choice. Several carers described proactively naming the diagnosis to pre‐empt misjudgment. One participant explained, “I always tell people… please forgive him, he has this condition” (Wife, Mid‐VD&AD). Naming dementia allowed behaviors to be relabeled as illness‐related rather than intentional, rude, or careless, helping protect both the dignity of the person with dementia and carers’ social relationships. As one professional carer explained, “she's forgotten you not because she doesn't care, it's because she's ill” (Pro‐F, NOS).

Many carers reported increased empathy, accommodation, and shared experiences following disclosure: “I've never felt so accommodated” (Daughter, Mod‐VD). Increased public awareness was noted as enabling openness: “10 years ago, you won't talk about it, but now you can” (Son, Dec‐AD). Despite this, disclosure also carried risks. Some carers described social withdrawal following disclosure: “some people never came to see her after. They probably couldn't handle it” (Son, Dec‐AD). Some carers interpreted social withdrawal as discomfort rather than overt stigma, suggesting others “don't want to upset you by saying the wrong thing” (Wife, Dec‐AD&PDD). Avoidance was also attributed to others’ prior negative illness experiences: “they might have bad experience about it… I understand why they avoid us” (Son, Mid‐AD). Stigma operated through both explicit rejection and uncertainty, requiring carers to continually negotiate when and how dementia was disclosed.

### 3.5 Reconfiguring caregiving practices and expectations

This theme captures how changing understandings of dementia translated into practical, relational, and moral adjustments in everyday care. Caregiving was described as an ongoing process of reconfiguration, in which daily routines, family roles, and care expectations were progressively reshaped in response to dementia. Caregiving became a totalizing orientation rather than a discrete role. One carer reflected, “I wake up to think about supporting them… it's in all my life every day now” (Pro‐M, NOS), while others described how family life increasingly revolved around dementia:

“I need to let my sister live her life as well and not be the only one carrying this load.” (Grandson, Sev‐AD).

The centrality of dementia was reinforced by the unpredictability of care: “no two days are the same” (Son, Mod‐VD) and limited ability to anticipate demands. This constant uncertainty required carers to manage not only the needs of the person with dementia but also their own wellbeing: “take a good care of myself to survive caregiving” (Daughter, Dec‐AD).

Care responsibilities were frequently renegotiated within families, often accompanied by guilt and moral tension. Several carers described deliberately absorbing greater responsibility to protect others: “I can do it right, so I will. My brothers don't understand what it takes” (Daughter, Mod‐VD). Expectations regarding care were also shaped by gender norms and professional identity. Women and carers with medical backgrounds described disproportionate burdens, often becoming “de facto case managers” (Wife, Mid‐VD&AD): “my mom won't let any of her sons help, so it's just me” (Daughter, Mod‐AD). Others emphasized shared responsibility: “we do what we have to do, man or woman” (Son, Mid‐AD).

Decisions about care settings represented a particularly fraught point of reconfiguration. While residential care was sometimes recognized as necessary, it was often experienced as morally distressing. Keeping relatives at home was framed as moral obligation, with placement interpreted as abandonment: “if you move your parents… it's like you throw them out… it's only when there are no other options” (Pro‐M, NOS). Even when care needs exceeded capacity, decisions were described as forced rather than chosen: “sooner or later, your hand is forced.” (Wife, Dec‐AD&PDD) One carer summarized:

“There are no good options, whether I keep her at home and lose my own life, rely on in‐home care that drains our savings, or place her in a nursing home and be the bad child.” (Son, Dec‐AD)

### 3.6 Carers’ unmet needs and support requests

Thematic analysis also identified practical support needs arising across the five themes, presented as a descriptive synthesis (Table 2).

**TABLE 2:** Key areas for support as identified by carers.

Category	Type of support needed	Illustrative quotes
**Family carers**		
Care support	Practical support	“Everyday things like grocery shopping, everything is just on me.”
		“I wish I knew how to keep them safe at home, how to manage hygiene.”
	Mental health support	“I need to manage myself so that I don't get angry.”
	Navigable information	“Understand what help you can get.”
		“There's a big gap. What do we do between diagnosis and nursing home?”
	Flexible peer‐ support	“Talk to people who've been through it.”
		“I can barely manage my responsibilities. How do I make time for support groups?”
	Ongoing, needs‐based training	“Someone that would sit me down and tell me what I needed to know.”
		“They should do assessments and refreshers every few years. Our needs changed.”
Care planning	Legal planning	“Get their Lasting Power of Attorney and will while you can. It's tough to do it when it's too late.”
	Financial planning	“We didn't plan ahead and had to mortgage our parents’ house for her nursing home fees.”
	Care continuity & adequacy	“It's hard to find a nursing home that suits your needs… I had one nearby and it closed down.”
		“They came in for 15 minutes now and then. I am here 24 hours a day.”
Healthcare system	Personalized care	“Care should be customized as much as possible.”
	Inclusive access (language & culture)	“If you don't fit the demographic, then you're out.”
		“She lost her English after a while, and by then we can't use the services anymore.”
		“My dad didn't grow up here… when they ask something very British, he can't answer.”
	Timely, joined‐up care	“She has more conditions other than dementia… nothing that joined the dots to see the whole person.”
		“We waited a very long time for appointments.”
	Quality assurance	“There should be far more scrutiny… to make sure patient safety is a priority.”
	Case coordination	“Having a case manager that we know and who knows us.”
**Professional carers**		
Workplace safety	Protection	“We got abused by the patients and family members.”
Ongoing support	Mental health support	“I had an anxiety attack because of this.”
Preparedness	Clinical training	“I don't have any medical background. I wish I got more training.”
	Health education	“I wish I could understand more about the condition and symptoms, so we are ready for what's to come.”
Workforce sustainability	Realistic job preparation	“It's a tough job. I wish I was told the heaviness of it when I started.”
	Burnout prevention	“I cared a lot at the beginning, but over time it got too hard. I just do it for the money now.”

## 4 DISCUSSION

This study highlights the iterative and relational nature of dementia caregiving among family and professional carers in the United Kingdom, encapsulated in the overarching “Re–” meta‐theme and five interrelated themes: re‐evaluating dementia, realigning acceptance and realities around diagnosis, reasoning with symptoms, revealing and relabeling dementia to the public, and reconfiguring caregiving practices. Carers navigated uncertainty, moral tension, and structural constraints while balancing competing responsibilities and sustaining the autonomy and dignity of the person with dementia. Disclosure and care‐setting decisions were central, shaped by cultural, generational, and ethical considerations, with carers adopting pragmatic strategies integrating personal, familial, and societal frameworks within the United Kingdom. Findings extend existing research by showing that caregiving involves ongoing cycles of reinterpretation and renegotiation rather than a single adjustment after diagnosis.

Our findings underscore the complex interplay between dementia, stigma, and caregiving preparedness. Carers initially distanced themselves from dementia, reflecting public misconceptions and low perceived susceptibility, consistent with reports of negative stereotypes, discrimination, and self‐stigma.^25^, ^26^ Awareness alone did not translate into preparedness, as carers often recognized dementia cognitively yet remained unready for the practical and emotional demands of caregiving. Jeyagurunathan^27^ similarly reported that carers recognized dementia and the need for diagnosis and treatment, but barriers such as denial, resistance from the person with dementia, and practical challenges can lead to unreadiness to seek help. Likewise, caregiving readiness depends on confidence, practical skills, and emotional resilience, with misinformation constraining carers’ ability to manage evolving needs.^28^, ^29^ Carter^30^ highlighted the role of targeted dementia education in improving preparedness through knowledge, anticipatory planning, and end‐of‐life discussions. Our findings further suggest preparedness should be understood as an ongoing process requiring continued education, emotional support, and adaptation as dementia progresses, with diagnosis marking the beginning rather than the endpoint of support.

Dementia remains heavily stigmatized, even among carers. Comparisons with cancer and depression revealed that dementia is perceived as more socially acceptable than mental illness but still subject to moral judgment and negative stereotypes. Cancer was viewed as a “brave battle” against a physical disease, whereas Alzheimer's disease was described as “being an old fart^30^”. Dementia frequently co‐occurs with depression and other mental health conditions,^31^, ^32^ amplifying stigma for care recipients and carers. People with dementia also face discrimination in employment, exclusion from medical decision‐making, and combined age‐ and disease‐related stigma,^33^, ^34^ reinforcing negative social perceptions carers must navigate. Public and systemic factors influenced caregiving decisions, including disclosure, seeking support, and institutional care use. These were often reactive, driven by visible symptoms, the desire to pre‐empt misjudgment, and cultural expectations.^35^ Nguyen^36^ found that carers worried about future dementia were more likely to priorities care planning, with knowledge of Alzheimer's disease increasing this likelihood. Harrison^37^ highlighted delayed care‐planning discussions by clinicians, illustrating how systemic gaps compound public stigma. Gender and professional norms further shaped caregiving, with women^38^ and medically trained carers disproportionately assuming primary responsibility. Although professional carers provided insights into stigma and structural constraints in formal care settings, their small representation limits interpretation; further research should explore their experiences and role in dementia care. Thus, targeted strategies are needed to improve public understanding of dementia and mental health, strengthen provider communication and care planning, and better support male carers to reduce gender disparities in caregiving burden.^39^

Carers employ a range of strategies to navigate the complex demands of dementia caregiving. Among some carers, faith‐based and spiritual interpretations provided a way to make sense of dementia, particularly when healthcare support was perceived as limited, and may support coping and meaning‐making.^40^ For these carers, the spiritual frameworks often coexist with medical care, enabling carers to manage uncertainty, accept the incurable nature of the condition, and sustain the autonomy and dignity of the person with dementia. Practical strategies also guided disclosure and care‐setting decisions, with carers balancing personal, familial, and societal considerations to protect relationships and cultural values. By integrating moral, relational, and pragmatic approaches, carers continuously reconfigured daily routines and responsibilities to meet evolving care needs.

### 4.1 Strengths and limitations

This study's strengths include purposive sampling of family and professional carers across diverse ethnicities, genders, dementia stages, and subtypes, allowing rich, in‐depth perspectives. Limitations include the small, non‐representative sample, particularly the small number of professional carers, limiting transferability across ethnic groups, dementia subtypes, care settings, and caregiving roles. UK‐based recruitment through research registers and community organizations may have introduced selection bias, as carers willing to discuss dementia may differ from those experiencing greater stigma or distress. The cross‐sectional design also limited exploration of caregiving trajectories over time. As with all qualitative research, findings reflect self‐reported experiences and are not intended to be statistically generalizable.

### 4.2 Conclusion

Dementia caregiving involves continuous reconfiguration of daily life, family roles, and care expectations, shaped by cultural norms, moral obligations, and structural constraints. Carers navigate uncertainty, manage evolving symptoms, and make complex decisions about disclosure and care settings while maintaining the autonomy and dignity of the person with dementia. These findings highlight the need for adaptive support systems that integrate carers into diagnostic and monitoring pathways, address healthcare disparities, and respond to the emotional, practical, and ethical demands of caregiving. Future research should evaluate the long‐term effects of disclosure and care placement strategies, develop culturally and socioeconomically tailored support programs, and conduct comparative longitudinal studies across healthcare systems to inform context‐specific interventions and policy for carers and care recipients.

## CONFLICT OF INTEREST STATEMENT

The authors declare no conflicts of interest. Author disclosures are available in the Supporting Information.

## ETHICAL APPROVAL

Ethical approval was obtained from the Health and Social Care Research Committee and the South Central—Oxford B Research Ethics Committee via the NHS Health Research Authority (REC reference: 25/SC/0149).

## CONSENT STATEMENT

All participants provided informed written or verbal consent.

## Supporting information



Supporting Information
